# Bridging Science and Care: 14 Years of Genetic and Biomarker Research in Neurodegenerative Diseases at an Argentine Memory Clinic

**DOI:** 10.1002/alz70855_103028

**Published:** 2025-12-24

**Authors:** Ezequiel Ignacio Surace, Leonardo Romorini, Mariela Marazita, Giulia Solange Clas, Victoria Massazza, Maria Florencia Alvarez, Daniel Avendaño, Tatiana Itzcovich, Horacio Martinetto

**Affiliations:** ^1^ Institute of Neurosciences (INEU), Fleni‐CONICET, Buenos Aires, Buenos Aires, Argentina; ^2^ FLENI, Escobar, Buenos Aires, Argentina; ^3^ Fleni, Buenos Aires, Buenos Aires, Argentina; ^4^ FLENI, Buenos Aires, Buenos Aires, Argentina; ^5^ Fleni, Escobar, Buenos Aires, Argentina; ^6^ Fleni, Buenos Aires, Argentina

## Abstract

Understanding the biological and genetic landscape of neurodegenerative diseases in diverse populations deepens our insight into complex pathophysiological mechanisms. Our laboratory, established 14 years ago in Buenos Aires, Argentina, serves as a regional hub for Latin America, focusing on familial and sporadic cases of Alzheimer's disease (AD), frontotemporal dementia (FTD), and amyotrophic lateral sclerosis (ALS). We were among the first in the region to incorporate cerebrospinal fluid AD biomarker analysis into clinical diagnostics. Our research includes genetic counseling, the study of novel genetic variants, and the development of patient‐derived cell models using induced pluripotent stem cell technology. In this talk, we will discuss the challenges of staying at the forefront of technological advancements and underscore the importance of international collaboration in advancing both local and global knowledge.

